# The Epigenetics of Aging in Invertebrates

**DOI:** 10.3390/ijms20184535

**Published:** 2019-09-13

**Authors:** Guixiang Yu, Qi Wu, Yue Gao, Meiling Chen, Mingyao Yang

**Affiliations:** 1 Institute of Animal Genetics and Breeding, Sichuan Agricultural University, Chengdu 611130, China; yugx1102@163.com (G.Y.); 18728153863@163.com (Q.W.); 18227553815@163.com (Y.G.);; 2 Farm Animal Genetic Resources Exploration and Innovation Key Laboratory of Sichuan Province, Sichuan Agricultural University, Chengdu 611130, China

**Keywords:** aging, crosstalk, DNA methylation, histone modification, ncRNA

## Abstract

Aging is an unstoppable process coupled to the loss of physiological function and increased susceptibility to diseases. Epigenetic alteration is one of the hallmarks of aging, which involves changes in DNA methylation patterns, post-translational modification of histones, chromatin remodeling and non-coding RNA interference. Invertebrate model organisms, such as *Drosophila melanogaster* and *Caenorhabditis elegans*, have been used to investigate the biological mechanisms of aging because they show, evolutionarily, the conservation of many aspects of aging. In this review, we focus on recent advances in the epigenetic changes of aging with invertebrate models, providing insight into the relationship between epigenetic dynamics and aging.

## 1. Introduction

Aging is an inevitable, time-dependent process in most living organisms, which involves functional decline, a steady increase in a plethora of chronic diseases, and ultimately death [1]. According to different biological scales, aging can be divided into “four layers”: (I) the organism’s decline in physical function and increased susceptibility to diseases; (II) systemic immune, metabolic and endocrine dysfunction; (III) cellular malfunction; and (IV) failure of biomolecular maintenance [2]. Epigenetics mainly acts in layers three and four, but also impacts other levels. Much research on aging has focused on genetic manipulation, and changing the activity of numerous genetic pathways can lead to lifespan extension in model organisms, for example, the insulin/Insulin-like growth factor-1) pathway (IIS), Target of rapamycin (TOR) signaling, Adenosine 5‘-monophosphate (AMP)-activated protein kinase (AMPK) and sirtuins [3]. However, recently more attention has focused on epigenetic changes, which have come to be considered one of the hallmarks of aging [4]. Chromatin structure is altered because of the loss of histone protein during aging [5]. There is global DNA hypomethylation during ontogenesis [6]. Most brain functions, including synaptic plasticity, learning and memory, decline with age when epigenetic changes occur, including changes in microRNA (miRNA) levels [7,8]. Studies have showed that aging in humans is also associated with epigenetic drift [9]. Model animals, such as *Caenorhabditis elegans* and *Drosophila melanogaster*, have been long used for the study of aging. Worms and fruit flies have natural advantages because of their short life cycles, being easy to house and feed, the power of available genetic manipulations, and the conservation of many mammalian aging signaling pathways. The average lifespan is 2 to 3 weeks for *C. elegans* at 20 °C, and 70 days for *Drosophila* at 25 °C [10]. Simple models have provided valuable insights into the aging process; for example, the IIS signaling pathway was first discovered in *C. elegans* and was later found to be conserved in both insects and mammals, where it regulates the rate of aging [11]. Therefore, this review will summarize recent advances in the roles of epigenetic changes in aging in these two invertebrates. 

## 2. DNA Methylation in Invertebrate Aging 

**Figure 1:**
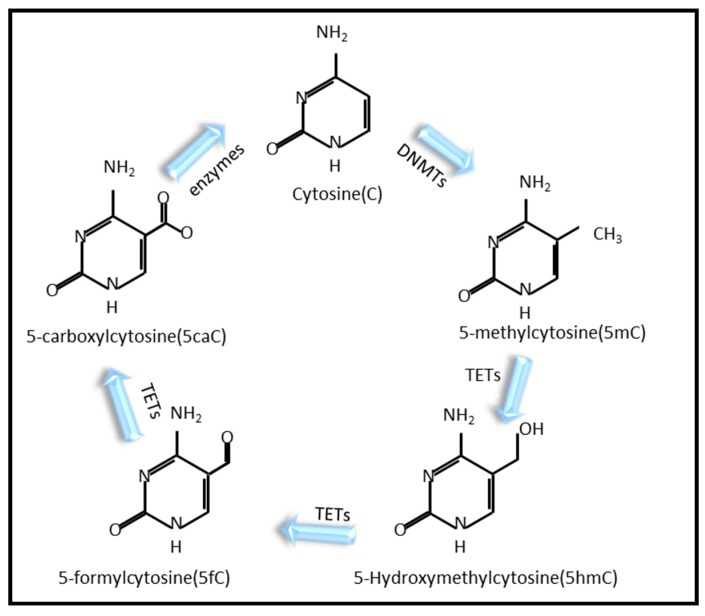
DNA methylation and demethylation. DNA cytosine methylation is catalyzed by DNA methyltransferases (DNMTs) to form 5-methylcytosine (5mC). Then, it can be oxidized iteratively by ten-eleven translocation (TET) protein to develop to 5-hydromethylcytosine (5hmC), 5-formylcytosine (5fC), and 5-carboxylcytosine (5caC), respectively. Finally, 5-carboxylcytosine (5caC) can be demethylated into the cytosine through the catalysis of a series of enzymes. Figure modified from Kohli and Zhang (2013) [17].

DNA methylation is a covalent chemical modification, which usually occurs at 5-methyl cytosine (5mC) and which is enriched in cytosine phosphate guanine (CpG) dinucleotides [12]. CpG methylation within promoters leads to transcriptional repression through the formation of compact chromatin structures such as heterochromatin. Conversely, promoters of genes that are highly expressed are devoid of DNA methylation, hence their name—CpG islands [13]. The dynamic changes in DNA methylation can impact aging and health. The local methylation level increases while the global methylation level decreases with aging [14]. Age-related DNA methylation changes are also correlated with human age-related diseases, such as cancer and sarcopenia [15,16]. Cytosine methylation is catalyzed by three DNA methyltransferases (DNMTs): DNMT1, DNMT3a, and DNMT3b. Three ten-eleven translocation (TET) proteins initiate the specific demethylation of 5mC residues in DNA: TET1, TET2, and TET3 [17]. The schematic diagram of DNA methylation and demethylation is shown in Figure 1. Worms do not encode a conventional DNA methyltransferase to silence DNA repeats, which led to the prevailing view that DNA methylation does not occur in *C. elegans* [18]. Similarly, in adult *D. melanogaster*, only a low level of DNA methylation was confirmed [19,20]. In *Drosophila*, overexpression of the DNA methyltransferase gene, *dDnmt2*, extends lifespan in a small heat shock protein-dependent way [21]. However, although 5mC methylation is rare, methylation on N6 adenine (6mA) is prevalent in *C. elegans* and *D. melanogaster* [22,23]. Recent studies have confirmed that NMAD-1 (MT-A70 family) and DMAD (DNA 6mA demethylase, TET ortholog) are 6mA demethylases in *C. elegans* and *D. melanogaster,* respectively. DAMT-1 (AlKB family) is likely a 6mA methyltransferase in *C. elegans* [24]. NMAD-1 and DAMT-1 can regulate 6mA levels and control the epigenetic inheritance of phenotypes associated with the loss of the H3K4me2 demethylase *spr*-*5* [22]. The worm mutant of *spr*-*5* displayed a transgenerational increase in H3K4me2 and 6mA levels coupled with a transgenerational fertility defect [22,25]. The double mutant of *spr-5* and *nmad-1* accelerates the progressive fertility defect, while the double mutant suppressed the transgenerational fertility defect in the *spr-5* single mutant [22]. Furthermore, *spr*-*5, nmad*-*1* and *admt*-*1* can regulate 6mA; therefore, it is supposed that an appropriate level of 6mA may be necessary to maintain normal fertility. DMAD was required for *Drosophila* development because transheterozygous mutants were either embryonically lethal or died within 3 days post-eclosion. In the ovary, DMAD-mediated 6mA demethylation is correlated with transposon expression [23]. DMAD depletion in the *Drosophila* brain results in brain developmental defects by 6mA accumulation. It was found that 6mA dynamic regulation by DMAD coordinates with trithorax and polycomb-mediated epigenetic mechanisms [26]. However, there is no direct convincing evidence of a link between 6mA and aging so far, to which more attention should be paid. 

## 3. Histone Modifications in Invertebrate Aging 

**Figure 2:**
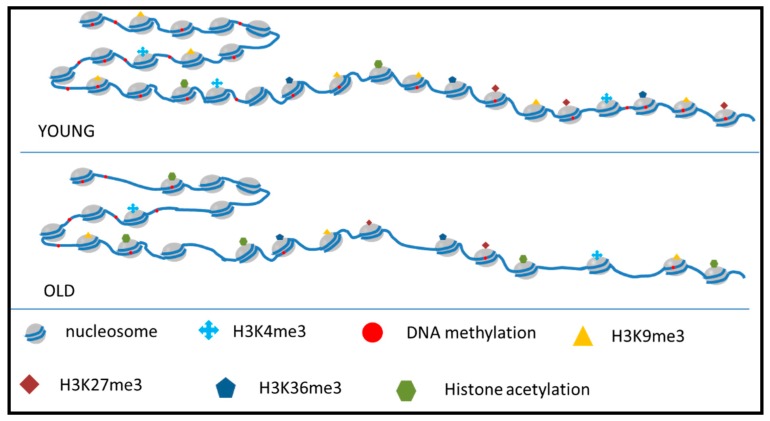
Histone modification changes during aging. During aging, DNA shows hypomethylation. A global loss of histone protein makes the chromatin structure looser. Histone methylation and acetylation show dynamic changes with age. These changes work together to contribute to the aging process.

Nucleosomes, the basic structures of eukaryotic chromatin, are made up of dimers of each core histone (H2A, H2B, H3 and H4). Histone modifications provide another layer of regulation beyond the DNA sequence itself. During the aging of organisms, the level of histone gradually decreases [5,13,27] but histone modifications show different changes. Histone modifications comprise several types, such as methylation, acetylation, phosphorylation and ubiquitylation [28]. Methylation and acetylation are thought to be most well-characterized modifying methods associated with aging [29]. Here, we will discuss those two types of modifications (Figure 2).

### 3.1. Histone Methylation in Invertebrate Aging

Histone methylation often takes place on lysine residues and is associated with aging in *C. elegans* and *D. melanogaster*. Like DNA methylation, histone methylation also requires methyltransferases and demethylases. Histone methyltransferases and demethylases are called KMTs/HMTs and KDMs/HDMs, correspondingly. We will review the roles of H3K4me3, H3K9me3, H3K27me3 and H3K36me3 associated with aging, respectively.

#### 3.1.1. H3K4me3

H3K4me3 is an epigenetic chemical modification involved in the regulation of gene expression. H3K4me3 showed diverse changes with age in worms. The canonical pattern surrounding the transcriptional start sites, which marks the 5′ end of genes [30], is established at an early stage during development and then remains stable with age. Non-conventional H3K4me3 regions preferentially mark gene bodies and are acquired during adulthood, and they further show age-dependent changes, which increase with age [31]. There are three major complexes responsible for generating H3K4me3 in mammals: the COMPASS complex, the Trithorax complex and the Trithorax-related complex [32]. Deficiencies of *ASH-2*, *WDR-5*, and *SET-2* (H3K4 methyltransferases in the COMPASS complex) reduce global H3K4me3 levels at the larval L3 stage and extend worm lifespan [33]. Knockdown or mutation of *RBR-2* from H3K4me3 demethylase was able to increase the level of H3K4me3 and then decrease lifespan. This affects the lifespan of animals in a germline-dependent manner [33]. Histone methylation has been implicated in transgenerational epigenetic regulation in *C. elegans* [34]. The loss of *WDR-5.1* or *SET-2* in worms impairs the transmission of stress adaptation in the progeny [35]. 

**Figure 3:**
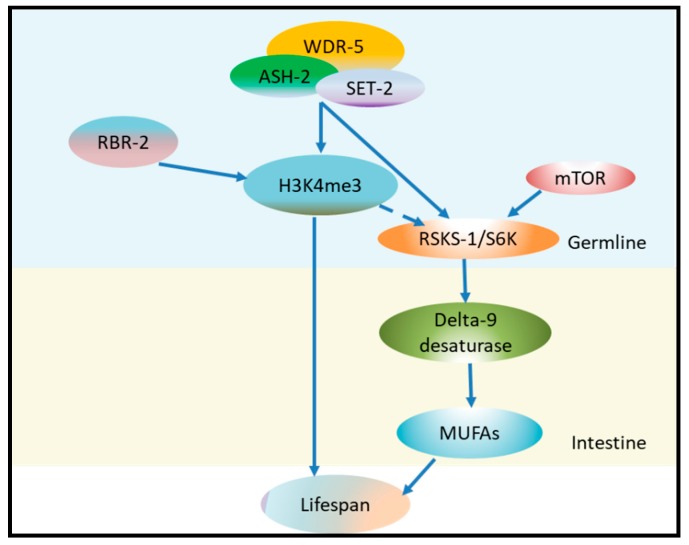
H3K4me3 regulates worm lifespan in a germline-dependent manner. RBR-2 demethylates H3K4me3, and the mutant of RBR-2 promotes the level of H3K4me3 and decreases lifespan. WDR-5, ASH-2 and SET-2 are the H3K4 methyltransferase complex, and the mutant in each one of them can diminish H3K4me3 level and prolong lifespan. The complex also directly or indirectly (dotted line) targets RSKS-1/S6K (a key conserved substrate of mTOR complex 1), which upregulates the delta-9 desaturase in the intestine, leading to mono-unsaturated fatty acid (MUFA) accumulation and extension of lifespan.

It was reported that there are still links between chromatin modifiers and fat metabolism. In *C. elegans*, the COMPASS H3K4me3 methyltransferase deficiency extends lifespan and promotes fat accumulation with a specific enrichment of mono-unsaturated fatty acids (MUFAs) in the intestine, by upregulating delta-9 fatty acid desaturase. This process acts mostly in the germline to regulate intestinal fat accumulation and lifespan, implying a germline-to-intestinal communication [36]. Importantly, these complexes can target RSKS-1/S6K in the germline, which is a key conserved substrate of mTOR complex 1 [37]. This suggests that the histone modification can act on mTOR signaling pathways to extend lifespan (Figure 3). 

A connection between metabolism and epigenetics also exists in *Drosophila*. Reduced levels of some enzymes involved in methionine metabolism disrupt its metabolism, which directly affects histone methylation levels. For example, the reduction of little imaginal discs (LID), the H3K4me3 demethylase, can counter the effects on histone methylation due to reduction of SAM-S (*S*-adenosylmethionine synthetase) [38]. However, the effect of LID in *Drosophila* lifespan is sex-specific, as the male *Drosophila* is more sensitive to the loss of LID-dependent H3K4me3 demethylation than the female [39]; however, the molecular mechanism for this remains unclear. Hcf (the *Drosophila* homolog of Hos cell factor 1) associates with the histone H3K4 methyltransferase Trithorax-related (Trr) to maintain H3K4 mono- and tri-methylation, regulating the Hippo pathway, which controls tissue and organ size through the regulation of cell proliferation and apoptosis [40]. 

Mutations in KDM5, another H3K4me3 demethylase, contribute to cognitive defects in flies and humans [41,42]. KDM5 can also regulate component genes of the immune deficiency (IMD) signaling pathway and maintain the host-commensal bacteria homeostasis in a demethylase-dependent manner. It was recently shown that a *Drosophila* mutant deficient in *kdm5* displayed gut dysbiosis, abnormal social behavior, and aberrant immune activation, and these phenotypes can be improved by *Lactobacillus plantarum* administration. This suggested a link between the gut microbiome and intellectual disability patients [43]. From all these results, H3K4me3 might act as a pro-aging factor to regulate lifespan. 

#### 3.1.2. H3K9me3

H3K9me3 is a further covalent methylation of histone protein. One of the HMTs that has been identified in *C. elegans* is histone-modifying enzyme MET-2, which targets H3K9. It was reported that MET-2 associates with a conversed DNA repair protein SMRC-1, which limits DNA damage and promotes DNA replication and repair and genome stability in the germline in worms [44]. Recently, JMJD-1.2 was found to have a demethylase activity towards several lysine residues on Histone 3 (H3) in *C. elegans. Jmjd-1.2* is expressed abundantly in the germline, where it controls the level of H3K9/K23/K27me2 both in mitotic and meiotic cells. However, *jmjd-1.2* mutants are more sensitive to replication stress, and the progeny of mutant animals exposed to hydroxyurea show increased embryonic lethality and mutational rate, which suggests a role for *jmjd-1.2* in the maintenance of genome integrity after replication stress [45]. Since genomic instability is one of the hallmarks of aging and is influenced by histone modification, we hypothesize that these hallmarks interact with each other during aging, making the mechanisms of aging more complicated. Associated with silenced heterochromatin regions, H3K9me3 serves as the binding site for heterochromatin protein 1 (HP1), which plays roles in heterochromatin formation, stabilization, and propagation [46]. In aged flies, the enrichment of H3K9me3 and HP1 was strikingly reduced. There is also an age-related change in the nuclear organization of H3K9me3 and HP1. Nuclei of fat body cells from young animals show a characteristic intensely concentrated staining for H3K9me3, while older animals show a more diffuse staining pattern [47]. Meanwhile, in the adult *Drosophila* mid-gut, the loss and dispersion of H3K9me3 and HP1 leads to the loss of chromatin stability in intestinal cells with age. Knockdown of *su(var)3-9*, methyltransferase for H3K9me3 or *HP1α* leads to intestinal stem cell (ISC) aging through genomic stress, JNK signaling, and apoptotic death in ECs [48]. The KDM4 family is highly conserved across species and reverses di- and tri-methylation of histone H3 lysine 9 (H3K9) and lysine 36 (H3K36). In *Drosophila*, Kdm4 is necessary for development because loss-of-function mutants do not survive past the early second instar larvae stage [49]. Apoptosis and DNA damage were involved in lethality [49]. Shown by the above evidence, H3K9me3 may contribute to DNA damage and genomic instability to promote the aging process.

#### 3.1.3. H3K27me3

Like H3K9me3, H3K27me3 is another typical epigenetic mark, which usually denotes transcriptional silencing. H3K27me3 levels increased in aged flies [50]. Polycomb repressive complex 2 (PRC2) is a multiprotein complex that catalyzes the methylation of H3K27. In *Drosophila*, heterozygous mutations in *E(z)* and *ESC*, both being core subunits of PRC2, increase longevity and reduce adult levels of H3K27me3 [51]. Mutations in *trithorax (trx)*, which is an antagonist of polycomb silencing and a methyltransferase for H3K4me3, elevate the H3K27me3 level of *E(z)* mutants and suppress their increased longevity. The mutants in *E(z)* and *esc* exhibit increased resistance to oxidative stress and starvation, and these phenotypes are suppressed by *trx* mutations too [51]. These results suggest that the H3K4me3 Trx complex and the H3K27me3 PRC2 complex may work together to regulate animal lifespan. Metabolic homeostasis is intimately connected with aging and lifespan regulation [52]. A reduction of H3K27me3 by PRC-deficiency promotes healthy lifespan in a glycolysis-dependent manner, as perturbing glycolysis diminishes the pro-lifespan benefits mediated by PRC-deficiency [50]. The *C. elegans* SET domain protein MES-2, an ortholog of E(z) (a subunit of PRC2), provides H3K27 methylation activity [53]. RNAi against *mes-2* extends lifespan significantly in wild type *C. elegans.* This also shows that the mechanism of life extension is independent of the germline [54].

The protein complex UTX-1 is a type of histone demethylase specific for H3K27me3, and it is a marker linked to chromatin repression. Recent work has shown that KDM6A/UTX is the target of metformin in prolonging lifespan through altering global H3K27me3 levels in mice [55]. The mutant of *utx-1* in flies raises the H3K27me3 level, limiting lifespan by downregulating glycolytic genes [50]. However, global somatic H3K27me3 levels decrease with age in germline-deficient worms [56]. RNAi of the *utx-1* gene extends the mean lifespan of *C. elegans* by 30% [57]. Meanwhile, both knockdown and heterozygous mutations of *utx-1* extend lifespan and increase the global levels of the H3K27me3 mark in worms [56]. Unlike H3K4me3, which extends lifespan mostly in a germline-dependent manner, H3K27me3 demethylase UTX-1 regulates lifespan independently of the presence of the germline, but in a manner that depends on the insulin-FOXO signaling pathway [56]. The catalytic enzyme utx-1 has an opposite effect on lifespan in flies and worms because it targets different genes in regulating lifespans. Most importantly, this phenomenon is mainly accomplished by different change patterns of H3K27me3 in aged flies and worms, increasing in aged flies but decreasing in aged worms. This suggests that only the optimal levels of H3K27me3 can maintain a healthy lifespan. 

#### 3.1.4. H3K36me3

H3K36me3 is a histone modification associated with active transcription, which plays crucial roles in a wide range of biological processes. A number of enzymes catalyze H3K36me. Loss of *Rph1*, a K36me2/3 demethylase, increases H3K36me3 level and extends lifespan in yeast [58]. Deficiency in the methyltransferase *met-1* results in globally decreased H3K36me3, an increase in global mRNA expression change with age, and a shortened lifespan in *C. elegans* [59]. This indicates that global mRNA change level is negatively correlated with H3K36me3. H3K36me3 facilitates genomic stability via the promotion of DNA damage repair, both in DNA mismatch repair and double strand break pathways [60]. The suppression of spurious transcriptional initiation within the gene bodies involving a Set2/SETD2 (methyltransferase of H3K36) mechanism can ensure the fidelity of gene transcription [61]. While SET-18 is the H3K36 dimethyltransferase, a *set-18* worm mutant extends lifespan and increases oxidative stress resistance in a *daf-16*-dependent manner. The level of muscle-specific *set-18* is activated in aged worms (day 7 and day 11), attributable to the promotion of H3K36me2 and the inhibition of *daf-16a* expression; subsequently, longevity is shortened [62]. These results indicate that H3K36me3 and H3K36me2 have different roles in aging. H3K4me3 extends lifespan via maintaining transcription fidelity and genomic stability, while H3K36me2 causes limited lifespan through the IIS pathway.

### 3.2. Histone Acetylation in Invertebrate Aging

Histone acetylation on lysine residues is another common histone modification, which plays a very important role in longevity regulation due to the direct connection with transcription promotion. Similar to methylation, it also requires the involvement of many enzymes, such as histone acetyltransferases (HATs) and histone deacetylases (HDACs). It was found that levels of H4K12ac, H3K9ac and H3K23ac increased in the midlife of *Drosophila*, compared with younger animals [63]. Early exposure to some mild stresses can slow down the aging process and extend lifespan through epigenetic changes. Histone acetylation levels of worms are increased under mild heat stress and are maintained into old age [64]. The expression of immune and detoxification genes also increased after such heat stress [64]. Histone acetyltransferase CBP-1 and the chromatin remodeling SWI/SNF complex (switch/sucrose non-fermenting, also known as the BAF complex) act as epigenetic modulators of the long-lasting defense responses [64]. When there is a decrease in the histone H4K12-specific acetyltransferase *chameau*, aging-associated phenotypes (such as raised oxygen consumption and acetyl-CoA levels as well as associated transcriptome changes) are alleviated, and longevity is prolonged [63]. In the meantime, Acetyl-CoA is a key metabolite in the TCA cycle and a cofactor for the acetylation of lysine residues, lowering the activity of the acetyl-CoA-synthesizing enzyme ATP citrate lyase (ATPCL), and also promoting longevity and retarding aging-associated changes [63]. Indeed, histone acetylation patterns are susceptible to alterations in key metabolites such as acetyl-CoA and NAD^+^, allowing chromatin to function as a sensor of cellular metabolism [65]. This implies that basical metabolism could be coupled with the aging process via lysine acetylation. Recent evidence indicates that histone lysine acetylation is tightly involved in the control of learning and memory [66]. Loss of *dCBP*, an acetyltransferase catalyze H3K23 in *Drosophila*, can decrease H3K23ac levels and impair neuronal gene activation, resulting in defective courtship learning [67]. These results imply that acetylation at different lysine residues may have opposite effects on aging.

Members of the sirtuin family of NAD-dependent protein deacetylases and ADP ribosyltransferases have been studied extensively as potential anti-aging factors. Overexpression of SIR2, a member of the sirtuin family, extended lifespan in budding yeast [68]. *Sir-2.1* and *dSir2* are the homologs of *S. cerevisiae SIR2* in *C. elegans* and *Drosophila*, respectively. Increased expression levels of *Sir-2.1* and *dSir2* extend the lifespan of worms and flies. However, the effect of *Sir2* is variable because of differences in genetic background and the mutagenic effects of transgene insertions [69]. Further research revealed that the effects of increased *dSir2* expression on lifespan in *Drosophila* are dosage-dependent. Significant lifespan extension is observed when *dSir2* expression is induced between two- and five-fold [70]. This effect is tissue-specific, as overexpression of *dSir2* in the pan-neuronal cells or fat body extended lifespan, whereas induction in motoneuron or muscles did not [71]. 

*Rpd3* is a zinc-dependent histone deacetylase in *Drosophila* and a homolog of mammal HDAC1. Reduction or inhibition of *rpd3* extends longevity, increases energy storage and downregulates gene expressions of the IIS pathway [72]. There is an overlap between *rpd3* and IIS longevity pathways, as mutations in *rpd3* and *dfoxo* showed weakened stress resistance compared with *rpd3* single mutant flies [72]. Lifespan extension in *rpd3* mutant flies may overlap with the mechanism of extension seen in dietary restriction (DR). *dSir2* has been implicated in mediating the response to DR in metazoans. Flies with double mutations in *rpd3* and *dSir2* had a median lifespan shorter than control flies, while *rpd3* mutants lived longer [73]. Continued exploration has found a potential interaction between *rpd3*-mediated longevity and the protein synthesis regulator *4E-BP* (a downstream of the TOR signaling pathway), based on the reduced longevity for both *rpd3* and *4E-BP* mutants compared to the single mutants of *rpd3* [74]. Therefore, *rpd3* is associated with the IIS, DR and TOR signaling pathways to promote longevity.

## 4. Chromatin Alterations in Aging

Changes in DNA and histone modifications are finally shown in chromatin changes. During aging, loss of histone and heterochromatin causes the chromatin structure to loosen, resulting in loss of transcriptional silencing. Retrotransposable elements are silenced by anchoring heterochromatin. The loss of heterochromatin with aging also leads to increased expression of otherwise silent retrotransposons [13]. The resulting transcripts from the retrotransposable elements are reverse-transcribed into cDNAs, which reinsert elsewhere into the genome of old cells, leading to genomic instability [75]. It was reported that HP1 (heterochromatin protein 1) destabilization was found in aged *Drosophila*, and overexpression of HP1 extended lifespan [76]. Deficiency of HP1α in enterocytes (ECs) leads to intestinal stem cell (ISC) aging, implying the loss of heterochromatin stability, which may be the crucial mechanism for ISC aging [48]. It seems that keeping heterochromatin stable could promote longevity.

It was known that SWI/SNF, NuRD (nucleosome remodeling and deacetylase) and the polycomb system make a difference in chromatin regulation [77]. Polycomb complexes are associated with chromatin containing repressive marks and silent or low transcriptional states [78]. Studies have pointed to a genetic antagonism between the SWI/SNF complex and polycomb repressive complexes 1 and 2 (PRC1/2) in *Drosophila*. Deletion of the BAF ATPases catalytic subunit *Smarca4* in mouse embryonic stem cells can cause a genome-wide increase in the localization of PRC1 and PRC2 and the abundance of H3K27me3, resulting in chromatin regulation [78]. The deficiency in CHD3, a subunit of the NuRD complex [79], elevated p53-dependent germline apoptosis, mainly due to the failure in the timely repair of double-stranded breaks, eventually leading to an increase in chromatin defects and apoptosis in worms [80]. In short, SWI/SNF contributes to the open chromatin state and active transcription; NuRD promotes the production of the repression of chromatin; and the polycomb complex promotes the formation of chromatin compression environments. 

## 5. Non-Coding RNAs in Invertebrates during Aging

Non-coding RNAs (ncRNAs) comprise various RNA species, including microRNA (miRNA), tRNA-derived small RNA (tsRNA), ribosomal RNA (rRNA), piwi-interacting RNA (piRNA), circular RNA (circRNA), and lncRNA [81]. ncRNAs have a remarkable impact on gene expression and chromatin remodeling by binding to their targets [82]. Mostly, total miRNA and piRNA are gradually decreased during aging in *C. elegans*, whereas in contrast, tsRNA, rRNA and circRNA levels generally display age-dependent increases [81]. The genetic modulation of specific ncRNAs affects longevity and aging rates by modulating established aging-regulating protein factors [81]. Generally, miRNA affects the aging process by acting on specific genes and altering their expression. miRNAs specifically target the 3′-UTR of mRNAs to exert transcriptional repression. A highly conserved miRNA, *miR-124*, was significantly upregulated in APS-induced longevity of *C. elegans* by regulating ATF-6 (an endoplasmic reticulum stress-regulated transmembrane transcription factor) [83]. The specific overexpression of the miRNA *let-7* in the *Drosophila* nervous system increased female median fly lifespan by ~22% [84]. RNA polymerase III can generate non-coding RNAs, including tRNAs. A reduction in RNA polymerase III can extend lifespan in worms and flies [85]. Therefore, this sheds light on tRNAs’ possible involvement in the aging process. 

In animals, piwi-interacting RNAs (piRNAs) of 21–35 nucleotides in length silence transposable elements; nearly all animals rely on piRNAs to defend the germline genome from transposon expression [86]. Piwi proteins combine with pi-RNA to silence targets post-transcriptionally. In flies, piwi promote H3K9 methylation, a repressive chromatin mark, through the recruitment of Eggless (also known as dSetdb1) by the piwi-interacting mediator proteins Asterix and Panoramix [87]. Piwi can repress heterochromatin loss, age-related dysfunction and apoptosis in ISCs, subsequently maintaining somatic stem cell genomic integrity [88]. 

CircRNAs are stable because the lack of free 5′-and 3′-ends protects them against nuclease attack [89]. These circRNAs can function as microRNA sponges to regulate mRNA, ultimately changing gene expression [90]. The expression of *circ_0005230* was elevated in human tumors and cholangiocarcinoma (CCA) cells, and it significantly facilitated cell growth, clone-forming ability and metastatic properties and inhibited cell apoptosis in CCA cells. Further study implies that *circ_0005230* could directly sponge *miR-1238* and *miR-1299* to exert its oncogenic function [91]. In humans, circRNA may also be involved in the regulation of ovarian function and aging by targeting certain miRNAs [92]. Therefore, specific circRNAs may function as anti-aging elements. 

LncRNAs with a variable length spanning from 200 bp up to several kilobases appear to be important for proper neurological functioning, with aberrant expression of lncRNAs leading to neurological disorders in *Drosophila* [93]. One study found that there is a high expression of the lncRNA *fer1l4* in human tumor tissues. Moreover, GO enrichment analysis also revealed that *fer1l4* may be involved in processes associated with tumorigenesis [94]. Recent research shows that lncRNAs are associated with organismal aging. The lncRNA *tts-1* in *C. elegans* extends lifespan, respectively, in *daf-2* (insulin/IGF-1 receptor) and *clk-1* (mitochondrial gene) mutations by reducing ribosome levels in a way that promotes life extension [95]. Therefore, some lncRNAs regulate lifespan in classic signaling pathways such as IIS pathway. 

## 6. Targets for Pharmacological Manipulation

**Figure 4:**
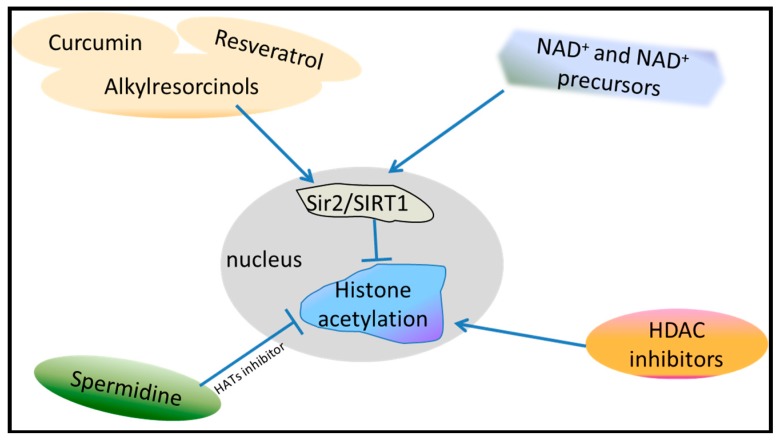
Epigenetic targets for pharmacological manipulation. Histone acetylation results in a more open chromatin state and greater access of DNA to transcription factors, leading to genome instability. Sir2/SIRT1 activators, such as curcumin, resveratrol and alkylresorcinols, can activate the sir2/SIRT1 activity to promote deacetylation. NAD+ and NAD+ precursors can also target sir2/SIRT1 to stimulate deacetylation. Spermidine, as a histone acetyltransferase (HAT) inhibitor, can suppress histone acetylation. Histone deacetylase (HDAC) inhibitors can promote histone acetylation.

Epigenetic markers have become particularly interesting because, in addition to acting as markers for the genetic regulation of aging, epigenetic mechanisms may be targets for drugs in aging and age-related diseases (Figure 4). Many drug trials have confirmed these proposals. Resveratrol, as an activator of Sir2/SIRT1 and AMPK, extends the lifespan of yeast [96], worms and fruit flies [97]. However, this lifespan-extension effect of resveratrol is abrogated by the *SIR2* mutation [97,98]. Natural compounds, such as curcumin or alkylresorcinols, enhanced SIRT1 activity and extended the lifespan of *Drosophila* [98,99]. NAD^+^ is a necessary cofactor for many metabolic pathways, such as glycolysis, fatty acid b-oxidation, and the TCA cycle. Also, NAD^+^ is also a substrate of sirtuin. NAD^+^ levels decline during aging across species [100,101], and supplementation of NAD^+^ extended the lifespan of worms, mice [102] and flies [103]. NAD^+^ precursors include nicotinamide (NAM), nicotinic acid (NA), tryptophan (Trp), nicotinamide riboside (NR), and nicotinamide mononucleotide (NMN); changes in these substances also affect sirtuins and then lifespan [104]. Therefore, Sir2/SIRT1 could be a promising target for aging intervention.

With aging, transcription levels of genes involving biosynthetic, metabolic and immune functions decline [105]. Histone acetylation promotes transcription activation, and deacetylation inhibits transcription, suggesting that pharmacological intervention in the process could impact longevity. Thus, HDAC inhibitors can increase longevity by promoting gene transcription. Inhibitors such as sodium 4-phenylbutyrate (PBA), sodium butyrate (SB), trichostatin A (TSA), and suberoylanilide hydroxamic acid (SAHA) affect several pathways involved in the regulation of these gene expression patterns associated with healthy aging [106]. Metformin, the first drug chosen to be tested in a clinical trial aimed at targeting the biology of aging per se, may exert its anti-aging effect by acting on H3K27me3 [55]. 

Spermidine, a naturally occurring polyamine, directly inhibits HATs, maintaining histone H3 in a hypoacetylated state in human cells. This altered acetylation status leads to significant upregulation of various autophagy-related transcripts, triggering autophagy in yeast, flies, and human cells, thereby enhancing longevity [107]. Spermidine can also block the age-related changes of cardiac cell composition and function, enhance diastolic function without affecting systemic blood pressure, and extend lifespan in an autophagy-dependent manner [108]. Spermidine or similar compounds related to HATs or autophagy could be good candidates to extend lifespan.

It is expected that drugs targeting epigenetic marks are the most promising for aging intervention.

## 7. Crosstalk between Epigenetic Marks

Epigenetic markers are involved in many physiological processes, and their interaction makes these processes more complex. It was shown that there was an inverse correlation between DNA methylation and histone H3K4 methylation in human cells [109]. Subsequently, genome-wide research found that DNA methylation could discriminate promoters from enhancers through H3K4me1-H3K4me3 in the seesaw mechanism [110], suggesting that the balance of seesaw might be used to determine whether the body is in a normal state. 

In many organisms, small RNAs modify chromatin via RNA interference pathways [111]. RNAi can produce long-term heritable responses that affect progeny [112]. In *C. elegans*, both exogenous and endogenous siRNAs can direct histone H3K27 methylation at targeted loci through the Nrde (nuclear RNAi defective) pathway, resulting in H3K27me3 status inheritance by progeny for multiple generations [113]. The induction of RNAi in *met-2* (H3K9 methylation enzyme) mutant worms resulted in the transgenerational barrier being broken, while RNAi was stably inherited transgenerationally; consequently, the progeny become progressively sterile due to the accumulation of small RNAs coupled by defective H3K9 methylation [114]. Therefore, ncRNA together with methylation could modulate transgenerational inheritance.

In the *Drosophila* brain, miR-34 becomes upregulated in an age-associated manner and is functionally required for lifespan extension, whereas mir-34 loss was shown to accelerate brain aging and degeneration and a decline in survival [115]. A more recent study revealed that miR-34 repressed the translation of *Pcl* and *Su(z)12* (two components of PRC2) transcripts, resulting in a reduction of PRC2 activity and less H3K27me3, promoting healthy aging [116]. Epigenetic marks’ interaction also exists in age-related diseases. In colon cancer cells, lncRNA CCAT2 acts as a negative regulator of miRNA-145 biogenesis, implying a novel mechanism of lncRNA-miRNA crosstalk [117].

There is also a crosstalk between histone deacetylase inhibitors and H3K4 methylation marks in prostate cancer cells [118]. It was reported that H3K9me3 and DNA methylations interact with each other, probably through HP1 [119]. In addition, enhance RNA (eRNA) is one of the non-coding RNA molecules [120], which can bind CBP at enhancers and stimulate histone acetylation and transcription of target genes [121]. The crosstalk between epigenetic marks is even more complex than that described above, thus making the aging mechanism more compelling.

## 8. Conclusions 

Epigenetic alteration involves changes in DNA methylation patterns, post-translational modification of histone, chromatin remodeling and non-coding RNA interference. These processes are each associated with the aging of *D. melanogaster* and *C. elegans*. However, we believe that the epigenetic landscape may be further complicated beyond the description above due to the crosstalk between epigenetic mechanisms. Understanding the epigenetic changes in the aging process can advance our knowledge of the mechanisms of aging. Drug research in epigenetics will be a powerful intervention in aging. A longer and healthier lifespan for humans could be achieved by leveraging the powerful genetic tools available for simple invertebrate models, in order to aid our understanding of aging mechanisms in the less tractable human system.

## Abbreviations

5mC	5-methylcytosine
6mA	N6-methyladenine
Acetyl-coA	Acetyl coenzyme A
AMPK	Adenosine 5‘-monophosphate (AMP)-activated protein kinase
ATPCL	ATP citrate lyase
CpG	Cytosine Phosphate guanine
DAMT	DNA adenine methyltransferase
DMAD	DNA 6ma demethylase
DNMT	DNA methyltransferase
DR	Dietary restriction
H3k27me3	Trimethylation of lysine 27 on histone H3 protein subunit
H3k36me3	Trimethylation of lysine 36 on histone H3 protein subunit
H3k4me2	Dimethylation of lysine 4 on histone H3 protein subunit
H3k4me3	Trimethylation of lysine 4 on histone H3 protein subunit
H3k9ac	Acetylation of lysine 4 on histone H3 protein subunit
H3k9me3	Trimethylation of lysine 9 on histone H3 protein subunit
HAT	Histone acetyltransferase
HDAC	Histone deacetylase
HDM/KDM	Histone demethylase
HMT/KMT	Histone methyltransferase
HP1	Heterochromatin protein 1
IGF-1	Insulin-like growth factor -1
IIS	Insulin/IGF-1 signaling pathway
ISC	Intestinal stem cell
MUFA	Mono-unsaturated fatty acid
NAD+	Nicotinamide adenine dinucleotide
NuRD	Nucleosome remodeling and deacetylase
PRC2	Polycomb repressive complex 2
SWI/SNF	Switch/sucrose non-fermenting
TCA	Tricarboxylic acid cycle
TET	Ten-eleven translocation
TOR	Target of rapamycin
